# Aerosol-Assisted Assembly of Mesoporous Carbon Spheres With Fast and Stable K-ion Storage

**DOI:** 10.3389/fchem.2020.00784

**Published:** 2020-09-08

**Authors:** Yu Guo, Jiahui Li, Hairui Wang, Limin Chang, Binglong Rui, Li Lin, Tianhao Xu, Ping Nie

**Affiliations:** ^1^Key Laboratory of Preparation and Applications of Environmental Friendly Material of the Ministry of Education, College of Chemistry, Jilin Normal University, Changchun, China; ^2^School of Materials Science and Energy Engineering, Foshan University, Foshan, China

**Keywords:** mesoporous carbon spheres, electrolyte, anode, aerosol spray, potassium ion batteries

## Abstract

Cost effective anode material with rational design is of significance for rechargeable potassium ion batteries (KIBs). Graphite anode currently still suffers unfavorable rate capability and moderate cycling stability. In this work, we report a mesoporous carbon sphere with rich porous structure as an anode material for KIBs with the assistance of an aerosol spray technology. The as-developed carbon spheres exhibit a well-defined spherical structure with favorable surface area of 1106.32 m^2^ g^−1^. Furthermore, the effect of different electrolytes on the electrochemical performance of the carbon anode has been investigated systematically. As expected, the carbon material shows excellent potassium storage performance in terms of improved specific capacity of 188.2 mAh g^−1^, rate capability and prolonged cyclability with a high capacity of 105.3 mAh g^−1^ after 500 cycles at a rate of 100 mA g^−1^ toward potassium storage in KFSI based carbonate electrolyte.

**Graphical Abstract:**
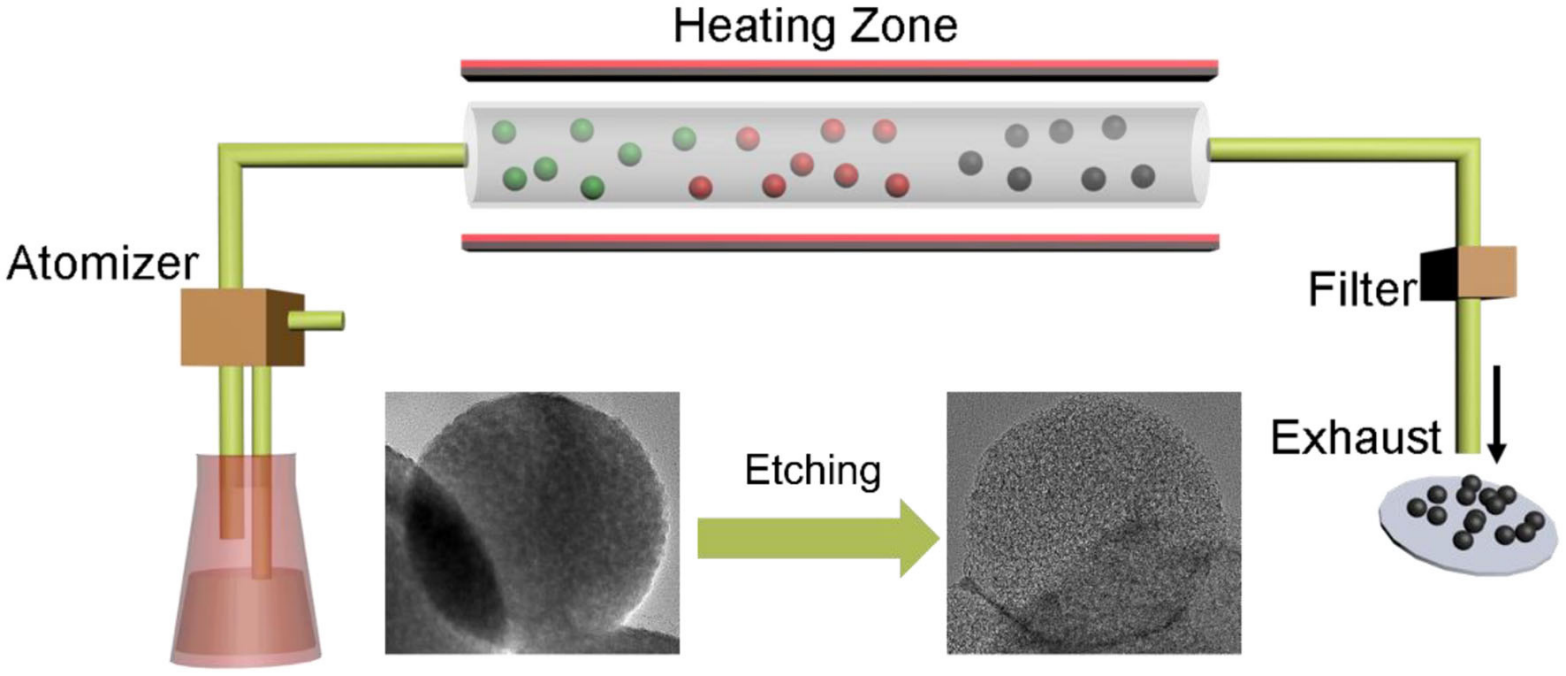
Aerosol-assisted assembly of mesoporous carbon spheres as anodes for K-ion batteries.

## Introduction

There are an ever-growing energy storage demands for high efficiency and low cost to power portable electronics, electric vehicles, and smart grid. In the past two decades, rechargeable lithium ion batteries (LIBs) have drawn significant attention due to their characteristics of high energy density, desirable cycle life and environmentally friendly (Peng et al., 2017; Liu Y. et al., 2019; Li X. et al., 2019; Sun et al., 2019; Wang F. et al., 2019; Chen et al., 2020). It is currently highly desirable to develop alternative battery systems with low cost and excellent cyclability to meet the increasing demand due to the limited resources of lithium and their uneven global distribution. Potassium locates within the same group in the periodic table with lithium, which exhibits similar chemical properties with the Li element. As a new energy storage device, potassium ion batteries show great potential for large scale application because of the abundant reserves of potassium resources in the earth's crust as well as a lower redox potential of K/K^+^ (−2.93 V vs. SHE) compared with Na/Na^+^ (−2.71 V), thus a wider potential window and higher energy density could be achieved (Le et al., 2017; Wu et al., 2017; Jiang et al., 2019; Li L. et al., 2019; Liu et al., 2019a; Zhang R. et al., 2019; Hosaka et al., 2020). Unfortunately, the relatively large radius of K^+^ (1.38 vs. 0.76 Å of Li^+^) leads to sluggish kinetics and poor cycling performance for most electrode materials in KIBs.

As compared to LIBs, the research on potassium ion batteries is still in its infant stage. Great efforts have been made to develop suitable host material with enhanced the K^+^ insertion/extraction kinetics. Currently, various electrode materials such as carbon materials (Hu et al., 2020; Liu M. et al., 2020; Zhang et al., 2020), alloys (Zhang et al., 2017; Lei et al., 2018; Zheng et al., 2019), metal oxide/sulfides (Li et al., 2020a; Liu Y. et al., 2020; Zhou et al., 2020), titanium based insertion materials (Sultana et al., 2016; Dong et al., 2018), and MXene (Okubo et al., 2018; Tang et al., 2020; Zhao et al., 2020) have been extensively studied as promising anodes for K-ion storage. Benefiting from the abundant resources, low cost, allotrope, and excellent physical/chemical stability, carbon materials have been widely reported and used for KIBs (Jian et al., 2015; Liu et al., 2019b; Zhang R. et al., 2019; Huang et al., 2020; Li et al., 2020b). The application of carbon based material for potassium ion storage requires high conductivity and large interlayer space for large K^+^ insertion. Commercially available graphite has been investigated as anode materials for KIBs with a theoretical capacity of 279 mAh g^−1^ based on reversible KC_8_ phase formation (Komaba et al., 2015; Luo et al., 2015). However, the material suffers from moderate cycling stability and unfavorable rate capability owing to large volume expansion (~58%) during K^+^ insertion. Therefore, the remaining issues existed in carbon materials for KIBs including limited reversible capacity and poor cycling stability need to be addressed.

Aerosol-spray shows great potential as a promising technology for the preparation of advanced functional materials for various application (Lu et al., 1999; Nie et al., 2018a). Aerosol-spray process mainly includes three steps: atomization, droplet to particle conversion, and product collection (Nie et al., 2017, 2018a,b). The synthesis begins with a liquid solution or suspension as a precursor, then desired material can be obtained through a simple and fast process, which is easy to produce electrode materials in scale. This method combines the advantages of both gas phase and liquid phase synthesis. By changing the process parameters such as precursor concentration, carrier gas flow rate, reaction temperature, and residence time, products with different sizes and morphologies can be prepared. Compared to other methods, aerosol-spray technology exhibits the great advantages in the preparation of materials for energy storage, where electrode materials with a rich porous structure and adjustable size can be obtained. The perfect structure obtained by aerosol is beneficial to ion transfer, increasing the electrolyte contact area, and accommodating volume change during cycling.

Herein, we have designed and fabricated a mesoporous carbon sphere (MCS) with rich porous structure as an anode material for potassium ion batteries with the assistance of an aerosol spray technology. Commercial available silica colloidal was chosen as a template because of its good dispersibility and nanosized particles with a size of 10–20 nm. The carbon sphere exhibits a well-defined spherical structure with favorable surface area. Furthermore, the effect of different electrolytes on the electrochemical performance of the porous carbon has been investigated. As expected, the mesoporous carbon shows excellent potassium storage performance in terms of improved specific capacity of 188.2 mAh g^−1^, rate capability, and prolonged cyclability in KFSI based carbonate electrolyte.

## Results and Discussion

**Figure 1:**
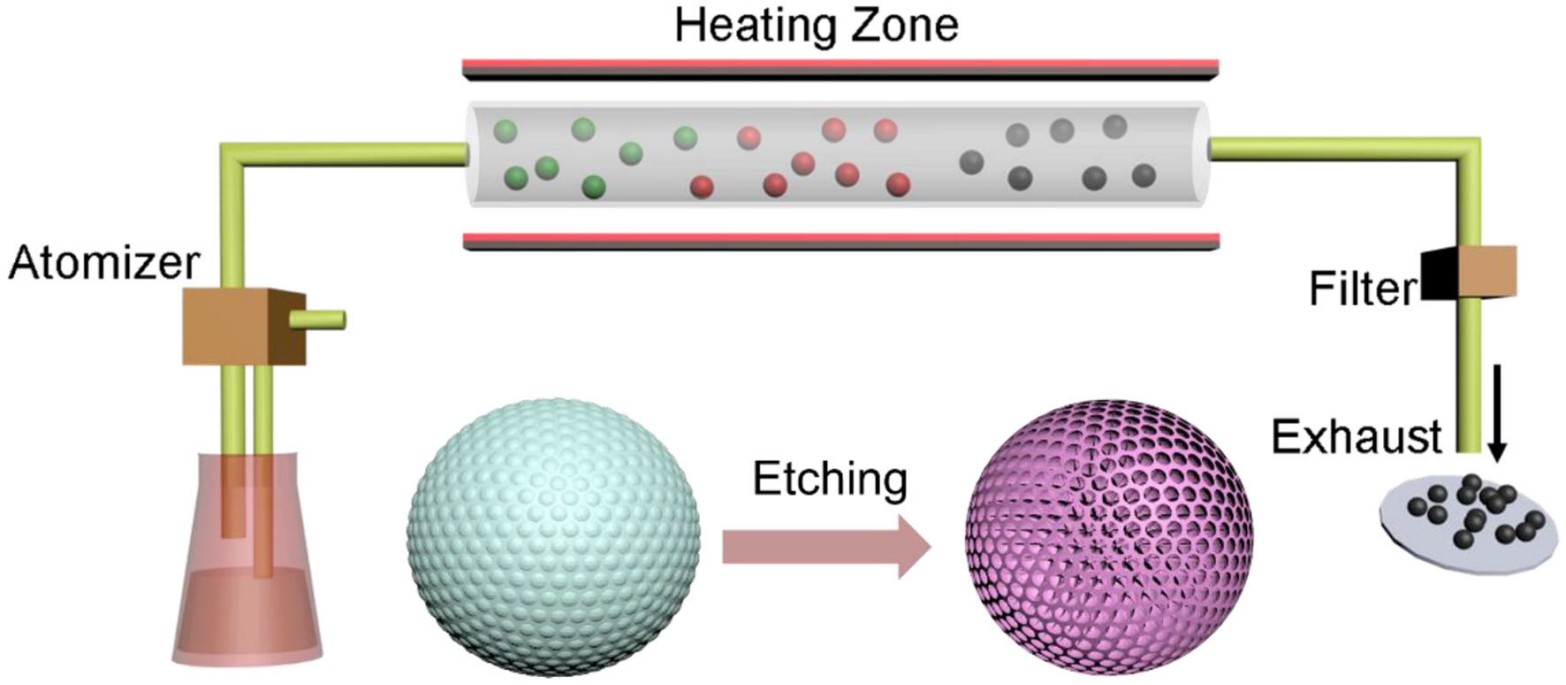
Schematic illustration of the preparation of mesoporous carbon spheres by aerosol-spray pyrolysis technology.

The mesoporous carbon spheres were prepared by a simple aerosol-spray pyrolysis technology as described in our previous work with colloidal silica and sucrose as precursors (Nie et al., 2017, 2018a,b; Liu X. et al., 2018), as shown in Figure 1. The precursor solution experienced an aerosol spraying process and subsequent carbonization under nitrogen flow at 900°C to derive the SiO_2_/carbon spheres. Then, the final mesoporous carbon spheres were obtained by removing the silica templates using dilute hydrofluoric acid (HF) etching.

**Figure 2:**
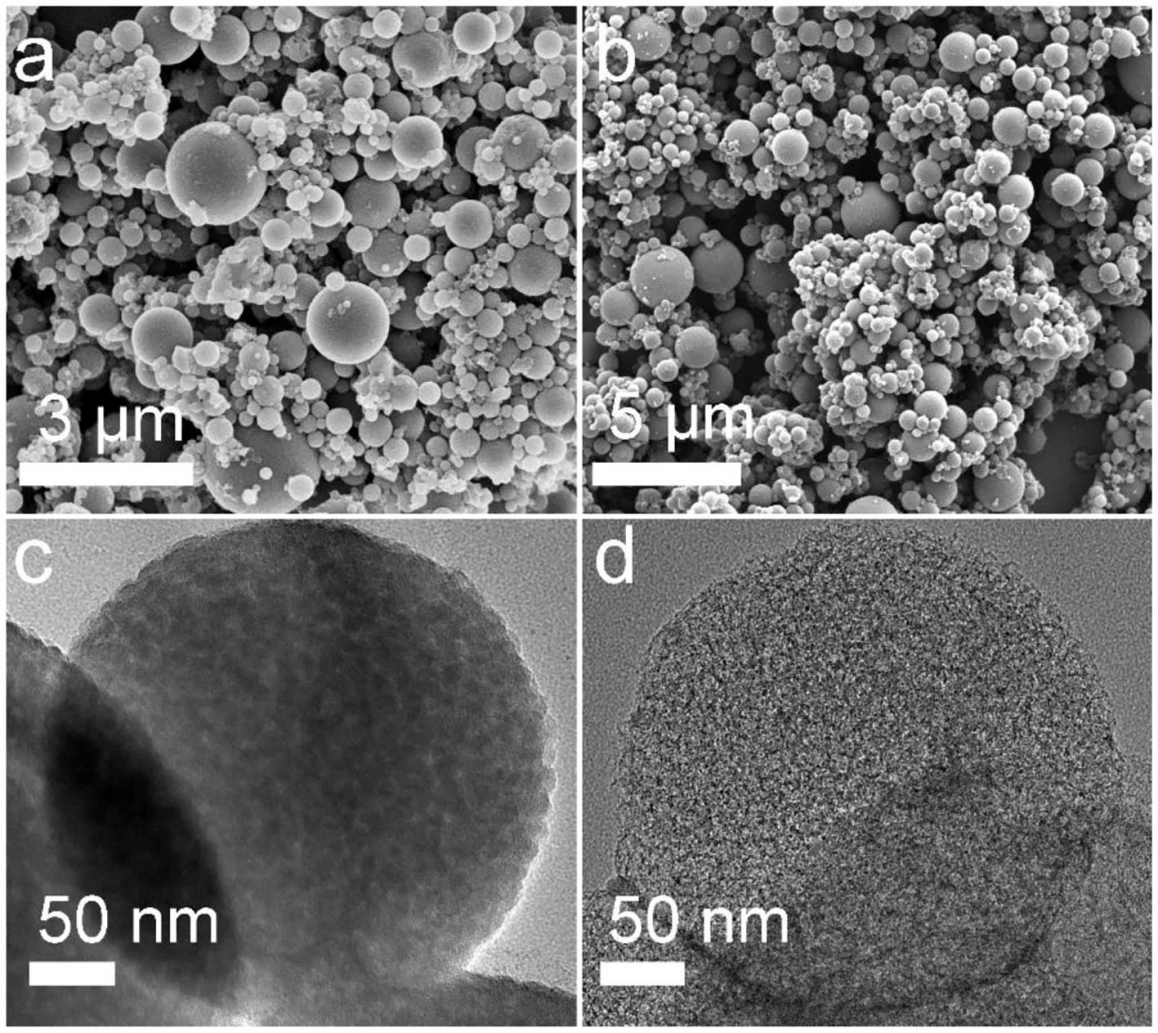
FESEM images of **(a)** the SiO_2_/C composite, **(b)** the MCS material; TEM images of **(c)** SiO_2_/C composite, **(d)** as-prepared MCS material.

Field emission scanning electron microscope (FESEM) and transmission electron microscopy (TEM) were performed to investigate the structural features of the as-prepared MCSs. It can be seen that well-defined spherical SiO_2_/C particles with a size ranging from 100 nm to 2 μm were successfully synthesized by aerosol spraying process followed by carbonization in an inert atmosphere (Figure 2a). TEM image of SiO_2_/C spheres confirms their typical porous structure consisting of small primary nanocrystals with an average size of *ca*. 10 nm (Figure 2c). After annealing at high temperature, the original spherical morphology was well-maintained with a negligible size change even upon acid etching (Figure 2b), indicating superior structure stability of the carbon particles. Further characterization shows the resulting carbon spheres exhibited more substantial porosity (Figure 2d), which was generated by the removal of the silica templates. Such a perfect porous spherical structure can not only increase the tap density of the carbon material, but also facilitate the fast ion/electron transfer, and effectively accommodate volume expansion upon K-ion insertion.

**Figure 3:**
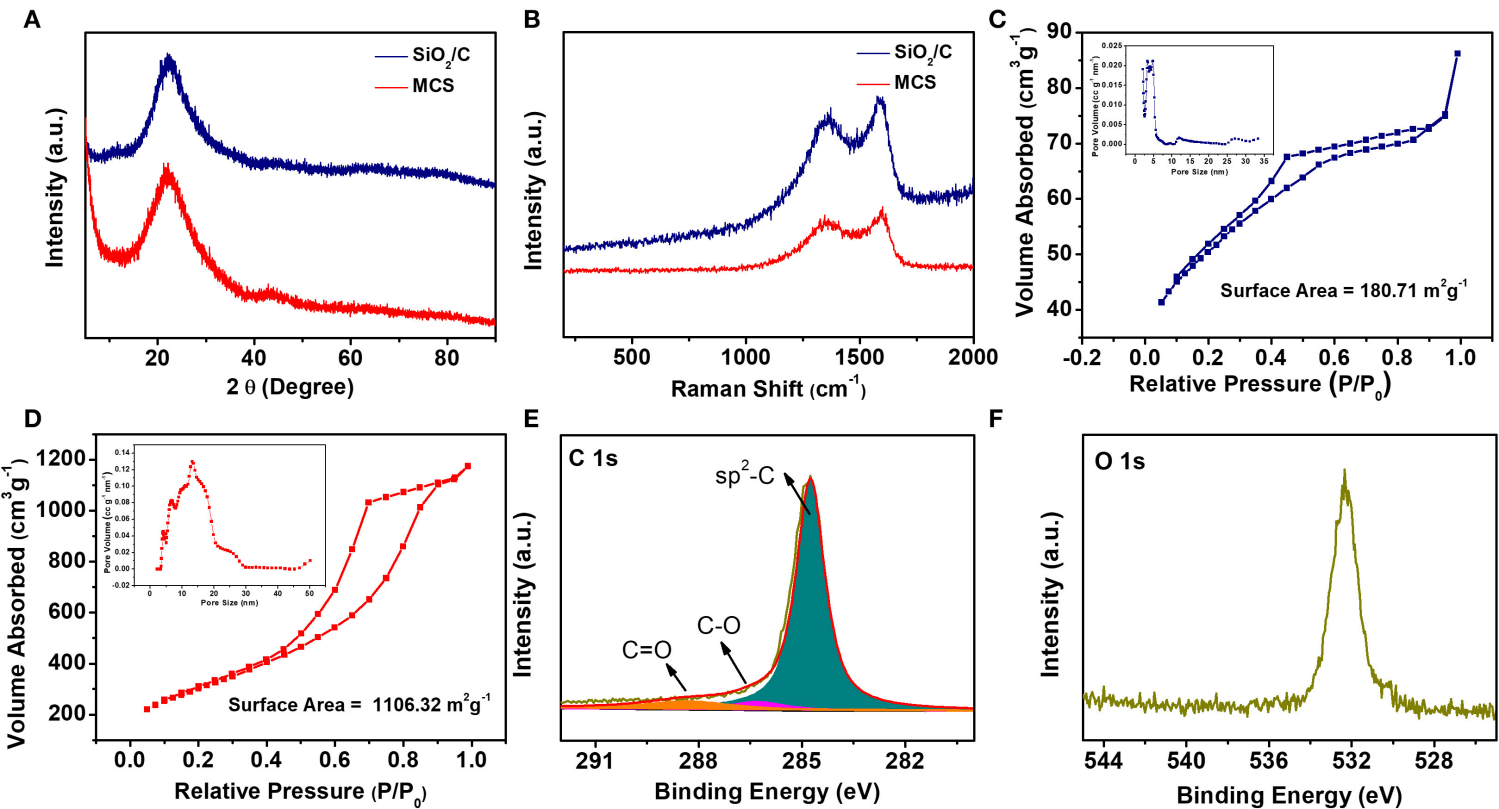
**(A)** XRD patterns and **(B)** Raman spectra of SiO_2_/C and MCS; N_2_ adsorption-desorption isotherms and pore size distribution of **(C)** SiO_2_/C and **(D)** MCS; XPS spectra of C 1s **(E)** and O 1s **(F)** for MCS.

To get further insight into the structure of the MCS, XRD patterns, and Raman spectra of the SiO_2_/C composite and the MCS were collected. As shown in Figure 3A, two broad peaks located at 22.1 and 43.3° can be observed for the MCS, which can be indexed to the (002) and (101) diffractions, typical characteristic peaks of carbon materials, indicating that porous carbon has been successfully obtained via the aerosol spray. Raman spectroscopy is another powerful and widely used tool to characterize the structural properties of carbon based materials. Figure 3B presents the Raman spectra of SiO_2_@C and MCS, respectively. For SiO_2_/C, two prominent peaks at 1,588 and 1,360 cm^−1^ are observed, corresponding to the well-documented G band (sp^2^ type graphitized carbon) and D band (sp^3^ type disordered carbon), respectively (Li et al., 2010; Hu et al., 2020). The Raman spectra of the MCS also contain both G and D bands. Notably, the peak intensity ratio (I_D_/I_G_) of MCS is calculated to be 0.88, suggesting the disordered carbonaceous structure.

The nitrogen adsorption-desorption isotherms of SiO_2_/C precursors and MCS are shown in Figures 3C,D. The Brunauer-Emmett-Teller (BET) specific surface area is calculated to be about 180.71 m^2^ g^−1^ for SiO_2_/C and 1106.32 m^2^ g^−1^ for MCS, respectively. The pore volume increases from 0.133 to 1.81 cm^3^ g^−1^ after etching. It found that etching away the silica template by HF greatly increases the specific surface area and pore volume of the carbon material. The SiO_2_/C exhibit pores centered from 2.1 to 33.2 nm, as shown in Figure 3C. Furthermore, the pore size of MCS is mainly distributed in 3.4–20 nm (Figure 3D, insert), demonstrating the mesoporous structure, which is well in accordance with the TEM result. The porous structure of MCS could significantly accelerate the permeation of electrolytes into the active material and shorten the diffusion pathway of potassium ion (Wu et al., 2019). X-ray photoelectron spectroscopy (XPS) was carried out to further characterize the surface chemical composition of the MCS. As shown in Figures 3E,F, the high resolution C1s spectrum of MCS reveal the existence of the C-C bond (284.8 eV), oxygen groups including C-O at 286.3 eV and C=O at 288.3 eV, respectively (Jayaramulu et al., 2018).

**Figure 4:**
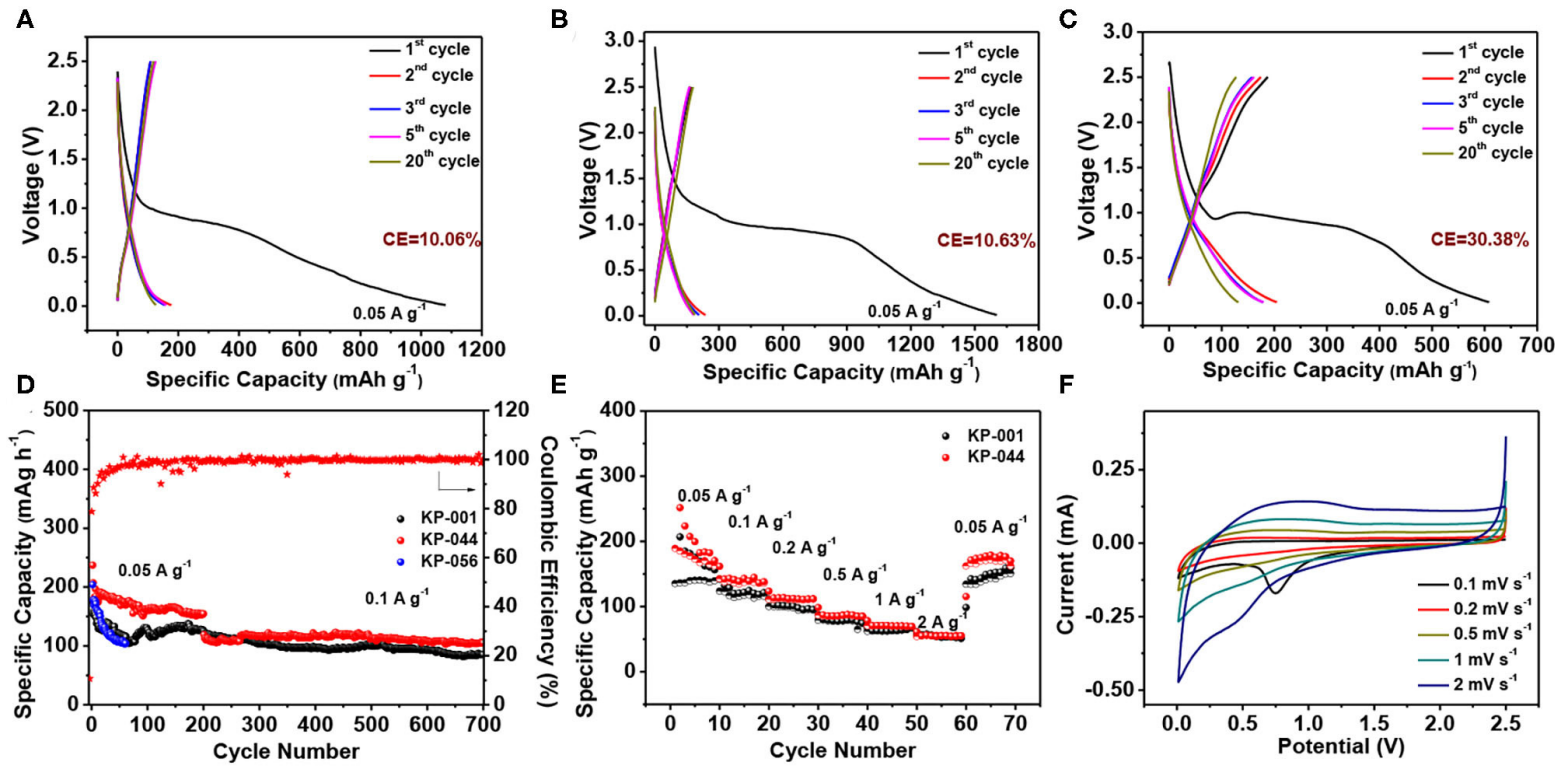
Electrochemical performance of the MCS anode under different electrolyte. The charge-discharge profiles at a current density of 0.05 A g^−1^: **(A)** KP-001, **(B)** KP-044, and **(C)** KP-056; **(D)** Cycling performance for KP-001, KP-044, and KP-056; **(E)** Rate performance for KP-001 and KP-044, **(F)** CV curves of MCS electrode with KP-044 electrolyte.

The electrochemical properties of the porous carbon materials were investigated in half-cells using metal potassium foil as both the counter and reference electrode. To evaluate the effect of different electrolytes on K-ion storage performance, three electrolytes including 0.8 M KPF_6_ in EC: DEC = 1:1 vol% (KP-001), 1.0 M potassium bis(fluorosulfonyl)imide (KFSI) in EC: DEC = 1:1 vol% (KP-044), and 1.0 M potassium bis(tri-fluoromethylsulfonyl) imide (KTFSI) in TETRAGLYME = 100 vol% (KP-056) were investigated for galvanostatic cycling. Figures 4A–C exhibit the galvanostatic discharge-charge curves of MCS in KP-001, KP-044, and KP-056 at a current density of 50 mA g^−1^. The initial discharge and charge capacities are 1080.4 and 108.7 mAh g^−1^ for KP-001, 1603.1 and 170.5 mAh g^−1^ for KP-044, 607.5 and 187.3 mAh g^−1^ for KP-056, respectively. It is remarkable to note that all of them present a long voltage plateau at 0.8–1.0 V, followed by a sloping curve down to the cutoff voltage of 0.01 V during the first discharge, which is a common phenomenon in carbon based anodes. The relatively large irreversible capacity loss can be related to the high specific surface area, causing irreversible electrolyte decomposition and the formation of solid electrolyte interphase (SEI) layer (Liu Y. et al., 2018), which leads to low Coulombic efficiency (CE). The first CE of MCS is 10.06, 10.63, and 30.38% in KP-001, KP-044, and KP-056, respectively. The reversible specific capacity in the second cycle is 125.5 mAh g^−1^ for KP-001, 188.2 mAh g^−1^ for KP-044, 130.7 mAh g^−1^ for KP-056. From the charge-discharge curves, we can conclude that the materials show good capacity retention in KP-001 and KP-044. Especially, the MCS exhibits the highest specific capacity in KP-044 electrolyte. After the 20th cycle, the discharge capacity is up to 178.9 mAh g^−1^, giving rise to a high CE of 95.1%.

As illustrated in Figure 4D, the porous carbon exhibits better cycling stability in KP-001 and KP-044 electrolyte. The discharge capacity is 124.9 and 154.5 mAh g^−1^ after 200 cycles at a current density of 50 mA g^−1^, respectively. High capacity of 84.9 and 105.3 mAh g^−1^ can be achieved after 500 cycle at a current density of 100 mA g^−1^, respectively. Compared with KP-001, KP-044 enables a higher capacity for the MCS anode, no obvious fading was observed over 500 cycles at 100 mA g^−1^. However, for KP-056 case, the carbon shows a fast capacity decay with a capacity of 104.4 mAh g^−1^ at 60th cycle. Notably, the rate capability of the MCS is also the best for KP-044. Figure 4E shows the rate performance of the MCS electrode at the current density from 50 mA g^−1^ to 2 A g^−1^. For the KP-001 electrolyte, the carbon material exhibits the capacity of 156.2, 121.2, 95.5, 74.5, 67.1, and 51.1 mAh g^−1^, respectively, compared to KP-044 with the discharge capacity of 170.5, 138.1, 111.6, 84.8, 69.1, and 55 mAh g^−1^ under the same current density. Furthermore, when the current density was returned back to 0.05 A g^−1^ after 60 cycles, the capacity could recover again, indicating the excellent reversibility of the MCS. The electrochemical performance of the mesoporous carbon in different electrolytes is also compared and summarized in Table 1. It should be noted that the potassium storage performance is comparable to those of many carbon based materials reported in literature (Table 2).

**Table 1:** Electrochemical performance of the MCS anode by using different electrolytes.

**Electrolyte**	**Initial CE**	**Specific capacity (mAh g**^****−1****^**) 50 mA g**^****−1****^			**Rate performance (mAh g**^****−1****^**)**		
		**2rd cycle**	**60th cycle**	**200th cycle**	**200 mA g^**−1**^**	**500 mA g^**−1**^**	**1,000 mA g^**−1**^**
KP-044	10.63%	188.2	174.2	154.5	111.6	84.8	69.1
KP-001	10.06%	125.5	114.4	124.9	95.5	74.5	67.1
KP-056	30.38%	130.7	104.4	–	–	–	–

**Table 2:** Electrochemical characteristics of reported carbon materials for KIBs in literature and our work.

**Anode materials**	**Specific surface area (m^**2**^ g^**−1**^)**	**Pore size (nm)**	**ICE (%)**	**Current density (mA g^**−1**^)**	**Specific capacity (mAh g^**−1**^)**	**Cycle life**	**References**
Ordered mesoporous carbon	1,089	0–20	63.6	50	257.4	100	Wang et al., 2018
Hierarchical porous carbon	604.4	0.2–8		50	211.5	50	Wu et al., 2019
CNTs/GCF	52.7	0–20	24	100	228	800	Zeng et al., 2019
Hard carbon	354	1.0–4.0	73	200	175	80	He et al., 2018
rGO				50	234		Luo et al., 2015
NHCNs	627			1,000	165	500	Liu et al., 2020
Crumbled graphene	318		39	40	340		Lee et al., 2020
OMFC-30			31.4	50	277	100	Zhang R. et al., 2019
HNCS	163.3		35	100	198	200	Sun et al., 2020
N-HCN	228.0			50	241	100	Ruan et al., 2019
Cellular N-C	465.3			100	279.3	200	Li et al., 2020b
N-CNS	654.42			1,000	369	500	Huang et al., 2020
SC-500	379.9	2.0	30	50	225.9	100	Tao et al., 2020
N-doped carbon nanosheets	827.5	1–100		100	210	450	Qin et al., 2019
MCS	1,106	3.4–20	10.63	50	154.5	200	This work

Figure 4F presents the CV curves of MCS electrode in KP-044 at different scan rate from 0.1 to 2.0 mV s^−1^ in the voltage range of 0.01−2.5 V. The CV curve shows a significant broad peak at around 0.75 V during the first cathodic scan and then disappears in the subsequent cycles, which is due to the decomposition of the electrolyte and formation of SEI layer. The sharp peak located at around 0.1 V is related to the insertion of K-ion into carbon (Hu et al., 2019). After the first cycle, the shape could be well-maintained with the increasing scan rate, indicating its excellent stability. Such good electrochemical potassium storage performance can be attributed to its unique porous architecture for the accessibility for electrolyte and electron transfer with enhanced conductivity. Moreover, the large specific surface area could provide sufficient space for K ion reaction at interface. Most importantly, previous reports have suggested that KSFI salt-based electrolyte could form a stable and robust SEI layer on electrode surface, and effectively suppress K dendrite growth and inhibit electrolyte decomposition to boost stable performance for K storage (Xiao et al., 2017; Wang H. et al., 2019).

**Figure 5:**
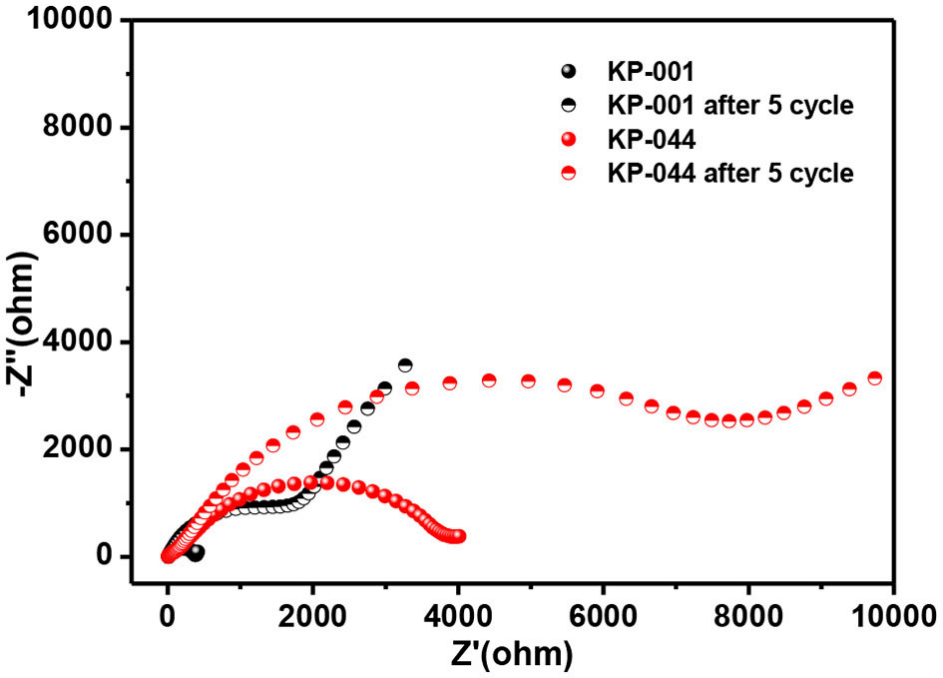
Nyquist plots of the MCS before and after 5 cycles using KP-001 and KP-044 electrolyte, respectively.

To further demonstrate the effect of different electrolytes, electrochemical impedance spectroscopy (EIS) was conducted in the frequency range from 100 kHz to 0.01 Hz. As shown in Figure 5, the Nyquist plots consist of a depressed semicircle in the high- and middle-frequency regions and a straight line in the low-frequency region, which correspond to the SEI film and contact resistance, charge-transfer resistance (*R*ct) and the diffusion of K-ion ions into bulk electrode, respectively (Li et al., 2018; Wang et al., 2018; Liu Y. et al., 2019; Zhang et al., 2019a,b). Figure 5 compares the EIS spectra of the carbon electrode before and after 5 cycles in KP-001 and KP-044 electrolytes. All the electrodes exhibit an increased *R*ct after cycling, consistent with the results reported (Li et al., 2018).

## Conclusion

In summary, mesoporous carbon spheres have been synthesized through a simple aerosol spray method by using low cost sucrose and silica colloid as precursors. The as-prepared porous carbon exhibited a well-defined spherical morphology with a size ranging from 100 nm to 2 μm. The elaborately designed nanostructure of the carbon spheres with high electrical conductivity and high surface area facilitates fast potassium ions and electrons transport, which make the mesoporous carbon sphere remarkable K storage property in terms of moderate reversible capacity, rate capability, and excellent cyclic stability. Furthermore, the KFSI electrolyte enables the porous carbon excellent electrochemical cyclability and improved capacity at high rate due to the formation of a stable and robust SEI layer. Encouragingly, the porous carbon material exhibited a high specific capacity of 154.5 mAh g^−1^ after 200 cycles at a current density at 50 mA g^−1^, and the capacity could be achieved at 105.3 mAh g^−1^ after 500 cycles at a rate of 100 mA g^−1^.

## Experimental

### Materials Synthesis

Typically, 15 g of colloidal silica (particles size: 10–20 nm) kindly provided by Nissan Chemical America Corporation (Houston, TX) was firstly mixed with 17 g 0.1 mol L^−1^ HCl aqueous solution. Then, 4 g sucrose was added to obtain a homogeneous solution under magnetic stirring. The precursor solution was next atomized by using nitrogen as a carrier gas with the heating zone maintaining at a constant temperature of 450°C. The collected samples were further annealed at 500°C for 5 h and 900°C for another 5 h under the N_2_ atmosphere with a heating rate of 10°C min^−1^ to obtained SiO_2_/carbon composite. The product obtained was subsequently washed with 5% HF and distilled water to remove the silica templates. After drying at 60°C in vacuum for 12 h, mesoporous carbon spheres were collected.

### Characterization

The crystal structure of the experimentally prepared porous carbon spheres was characterized by X-ray diffraction (XRD, Rigaku d/max PC2500) in the range of 5 to 90°. The morphology and microstructure characteristics of the samples were observed using a field emission scanning electron microscope (FESEM, JSM-7800F) and a high resolution transmission electron microscope (TEM, JEM-2100, JEOL). The pore size and specific surface area of the sample were analyzed using an Isorb-HP2 analyzer (Quantachrome Instruments) to measure the N_2_ adsorption/desorption isotherms at 77 K under liquid nitrogen. The X-ray photoelectron spectrum (XPS) was collected on a ESCALAB 250Xi spectrometer with a mono Al Kα radiation. The Raman spectra were tested by a Renishaw 2000 System.

### Electrochemical Measurement

For the electrochemical measurements, the as-prepared materials were mixed with acetylene black and sodium alginate in a weight ratio of 70:15:15. The mixture was prepared to form uniform slurry in deionized water and spread onto copper foil current collector by using a doctor-blade technique. After drying at 70°C in vacuum, the foil was roll-pressed and cut into circular pieces. Coin-cells were assembled in an argon-filled glove box using metal potassium as the counter electrode and Whatman® glass fiber as the separator. Three different electrolytes were used, including 0.8 M KPF_6_ in EC: DEC = 1:1 vol% (KP-001), 1.0 M KFSI in EC: DEC = 1:1 vol% (KP-044), 1.0 M KTFSI in TETRAGLYME = 100 vol% (KP-056). Galvanostatic charge/discharge cycles were tested on a cell test instrument (CT2001A, LAND Electronic Co., China) at a current density of 50 mA g^−1^ between 0.01 and 2.5 V. The specific capacity was calculated based on the weight of the mesoporous carbon spheres. Cyclic voltammetry (CV) was performed using a CHI 660E electrochemical workstation (CH Instruments, Chenhua, China) at a scan rate of 0.1 mV s^−1^ within the voltage range of 0.01–2.5 V. Electrochemical impedance spectra (EIS) were collected in the frequency range from 100 kHz to 0.1 Hz on the CHI 660E electrochemical workstation with a voltage perturbation of 5 mV.

## Data Availability Statement

The raw data supporting the conclusions of this article will be made available by the authors, without undue reservation.

## Author Contributions

YG, LC, and PN designed the experimental work and co-wrote the manuscript. YG and PN conducted the materials synthesis. BR, YG, and HW conducted the characterization. YG, JL, and LL performed the electrochemical measurements in potassium ion batteries and the related data processing. TX revised the manuscript. All authors analyzed the results and commented on the manuscript.

## Conflict of Interest

The authors declare that the research was conducted in the absence of any commercial or financial relationships that could be construed as a potential conflict of interest.
